# Issues Faced by Prosthetists and Physiotherapists During Lower-Limb Prosthetic Rehabilitation: A Thematic Analysis

**DOI:** 10.3389/fresc.2021.795021

**Published:** 2022-01-10

**Authors:** Shruti Turner, Athina Belsi, Alison H. McGregor

**Affiliations:** ^1^Centre for Blast Injury Studies, Imperial College London, London, United Kingdom; ^2^Sackler Musculoskeletal Laboratory, Department of Surgery and Cancer, Imperial College London, London, United Kingdom; ^3^Department of Surgery and Cancer, Imperial College London, London, United Kingdom

**Keywords:** amputation, rehabilitation, prosthetic rehabilitation, prostheses, lower-limb amputation, thematic analysis

## Abstract

Successful prosthetic rehabilitation is essential to improve the physical and mental outcomes of people with lower-limb amputation. Evaluation of prosthetic services from a prosthesis user perspective have been published and commissioned by the national bodies, however, the perspectives of clinicians working with service users during rehabilitation have not to date been sought. We sought to determine factors impacting lower-limb prosthetic rehabilitation from a clinician's perspective to inform studies focusing on prosthetic and socket design and fitting. Six clinician (2 prosthetists, 4 physiotherapists) interviewees were self-selected from a survey exploring issues and frustrations during lower-limb prosthetic rehabilitation. Semi-structured interviews explored the impactors on and frustrations with rehabilitation and the prosthetic socket. A thematic analysis was subsequently conducted to identify themes in the responses. Five themes were identified: Service Disparity, Body Impactors, Consequences of Ill-Fit, Prosthesis Irritants, and Limitations of Practice. Each theme, though distinct, relates to the others either as a cause or consequence and should be viewed as such. Addressing the themes will have benefits beyond the issues addressed but also expand into the other themes. This study provides an insight into the clinician perspectives on lower-limb prosthetic rehabilitation, which has not been formally documented to date.

## Introduction

Successful prosthetic rehabilitation of people with lower-limb amputations is vital to their ability to live and function independently. Some evaluation of prosthetic services from a prosthesis user perspective have been published in literature (1–3), and also commissioned by the National Health Service (NHS) (4). Issues such as the satisfaction and comfort with the prosthesis, difficulties with lifestyle adjustment and communication between clinicians and patients have been reported.

Throughout rehabilitation, patients interact with various clinicians, including occupational therapists and surgeons, amongst others. However, most of their interactions will be with prosthetists, who prescribe and fit the prosthesis, and physiotherapists, who guide strength training and movement re-education.

In most settings, after initial recovery from the amputation surgery the patient is discharged from inpatient treatment. Prosthetic rehabilitation is done primarily in an outpatient setting, with regular visits to the prosthetists and physiotherapists. Depending on the setting, prosthetists and physiotherapists may work in the same location and meet regularly to determine the best course of action for their patients. In other settings, they may be geographically separated and discuss care paths only as required e.g., if an issue with the prosthesis arises in a physiotherapy session.

Of the literature on the evaluation of prosthetic care, most is from the perspectives of the service users and based on questionnaire data (1–3). While questionnaires can provide an overview of the issues faced, due to generally larger responses than other methods, they are limited as detailed information is not collected. With multiple choice questions or scales, and short answer questions a large amount of the participants' experiences and reasoning are lost.

One study evaluated prosthetic service provision in the UK from the perspective of clinicians (5). However, this study focused specifically on clinician decision making during rehabilitation, rather than the experiences of and impact of prosthesis users.

This study aims to start to highlight the experiences of rehabilitation clinicians, and how they impact lower-limb prosthetic rehabilitation. The understanding of clinician experiences is important to gain a broader picture of the potential impactors on rehabilitation as they are involved in aspects that service users are not. Due to the clinician roles, they are part of many people's rehabilitation and therefore see a broader picture than an individual's personal experience and are able to draw trends from their practice.

## Materials and Methods

To explore the factors impacting rehabilitation first-hand, in particular socket-fit, a questionnaire with an optional follow-up telephone interview, approved by Joint Research and Compliance Office at Imperial College London (ICREC Reference: 18IC4485), was deployed for prosthesis users and rehabilitation clinicians. Both prosthesis users and rehabilitation clinicians were recruited for the surveys and interviews, however, in this paper only the interviews of rehabilitation clinicians are discussed. The analysis of the survey results and prosthesis user interviews have been published previously (6, 7).

The survey was created with input from rehabilitation physiotherapists as standard outcome measurement tools did not meet the needs of the study. The survey started with a free text question about rehabilitation frustrations to collect uninfluenced views, before questions being introduced specifically about sockets. The interview questions, however, were based on the responses given in the survey, related to any of the topics mentioned as having the most impact or being a particular frustration through rehabilitation.

At the end of the survey, participants could provide their contact details to participate in the telephone interviews (these were removed for analysis of data to preserve anonymity). The goal of the semi-structured interviews was to understand the reasons and impacts surrounding the answers given in the survey. Telephone interviews were chosen to minimize expenses and travel difficulties, facilitating higher participation.

The survey was created and shared online using Qualtrics, a web-based survey tool. The Participant Information Sheet was provided, followed by a checkbox to confirm participant consent, before the survey was displayed. The survey link was distributed via social media and emailed to personal contacts in relevant clinical teams, charities, and professional organizations relevant to the professions sought, to recruit relevant rehabilitation clinicians. Participants had to be 18 years or older; currently not taking medication that affected their cognitive function; have a good understanding of written and spoken English; and be a current member of a lower-limb prosthetic rehabilitation team.

All those who provided their details were contacted to complete the follow up interview, a total of eight clinicians completed the telephone interview. Recruitment was limited by the number of willing participants in the study period. Telephone interviews lasted no more than one h and were dictated by the length of answers given by participants. Relationships between participants and professional body membership were not monitored.

To analyse the issues experienced during rehabilitation, a thematic analysis was conducted on the interviewee responses. All transcriptions were anonymised before analysis. Steps for analysis were taken, based on Braun & Clarke's methodology: familiarization, coding, generating themes, reviewing themes, defining and naming themes, and reporting (8). Professional transcription of the interview recordings was completed, followed by coding by hand on printed transcripts and tabulation of key information. The codes were assessed and grouped to identify five distinct, but interrelated themes, and agreed by two researchers (ST and AM).

## Results

Of the 44 clinicians that completed the survey, eight UK based clinicians participated in the telephone interviews, however, two of the recordings were corrupted before the start of the thematic analysis. Therefore, the responses of six clinicians have been included in the presented results and discussion. Participants were varied in terms of years of experience and role, with two of the participants being prosthetists and the other four physiotherapists, and the vast majority being female (Table 1).

**Table 1:** Participant demographics, *n* = 6.

**Participant**	**Sex**	**Years experience**	**Work context**
Prosthetist 1	Male	12	Private
Prosthetist 2	Female	5	NHS
Physiotherapist 1	Female	28	NHS
Physiotherapist 2	Female	0.33	NHS
Physiotherapist 3	Female	20	NHS
Physiotherapist 4	Female	3	Private

**Figure 1:**
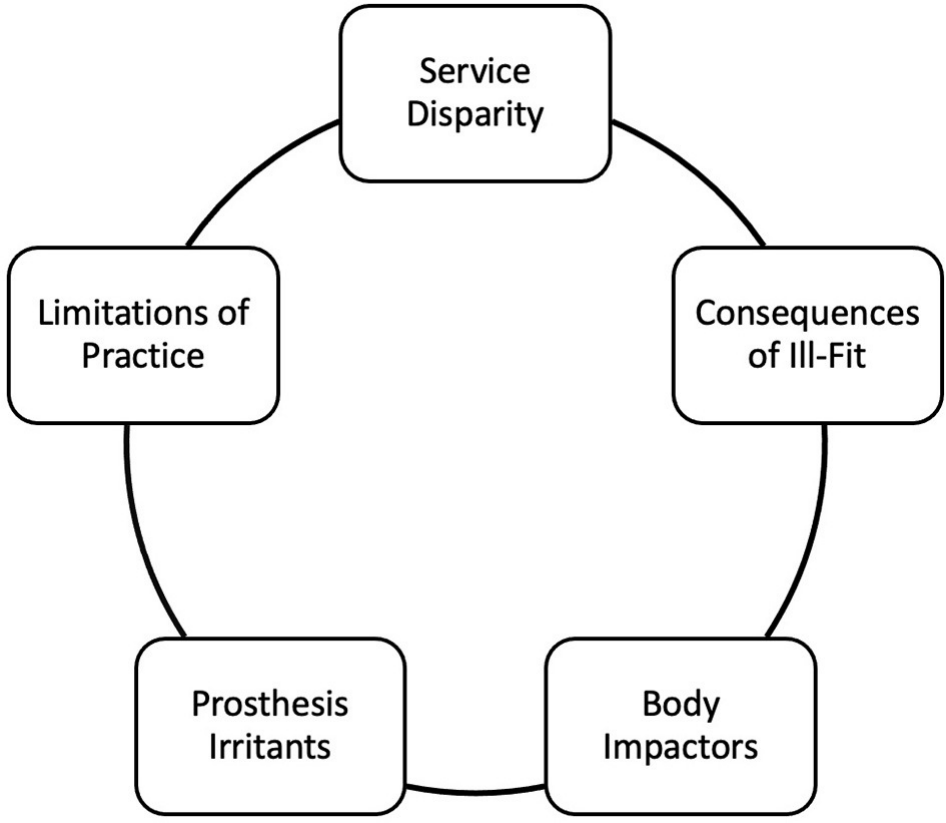
Themes identified from interviewee responses.

Five interrelated themes were identified (Figure 1): *Service Disparity, Body Impactors, Consequences of Ill-Fit, Prosthesis Irritants*, and *Limitations of Practice*. Quotations are accompanied by a role, participant number and sex to allow the range of views to be seen. The data was sex disaggregated for transparency, as females tend to be underrepresented in research though this is not the case in this study. Themes were not identified for or compared between the different professional roles due to the small sample size.

### Service Disparity

There are a variety of settings for prosthetic provision, including military rehabilitation centres with inpatient and outpatient care, NHS inpatient and outpatient care, and private clinics providing mainly outpatient care. Clinicians noted the *Service Disparity* between private and military settings, compared to the NHS regarding longer waiting times for appointments and socket refitting.

“I'm a bit different because I work privately; our sockets are generally a bit better” (Physiotherapist 4, Female)

“In the NHS, I guess it's worse as well-because you've got a bigger time (to wait for an appointment)…patients may be on that ill-fitting socket for six weeks, eight weeks whilst they get the appointment to get a new one started” (Prosthetist 1, Male).

Clinicians also noted a lack of continuity in care; the change in management and number of temporary clinicians could make it difficult for continuity of patient care. Time to update new clinicians on patient history means that there is less time to for clinicians to spend with patients or work on self-development to improve their practice.

“(Contracted clinicians) might change every…8–12 weeks…when I'm talking to the prosthetist about an issue for a patient, then you're obviously effectively starting again in terms of supplying them with information about the patient's…history… you're not necessarily getting the clinician knowing as much about the client as ideally, they would do” (Physiotherapist 3, Female).

Changes in the team had an impact on how different clinical teams worked together, and team setups varied in settings and locations. The settings where prosthetists and physiotherapists were in the same location was an advantage. They allowed for small issues with the prosthesis discovered during physiotherapy to be solved quickly. There was a view that the clinical teams that worked together when seeing patients, whether in the civilian or military environment, meant that patients could be treated and their issues solved with multiple perspectives considered.

“I think the biggest frustration in the NHS which I don't have so much (in the private setting) was time constraints; so, not being able to work jointly with the Prosthetists as well…the patient adapts and gets stronger with the physio there's a delay between the Prosthetist seeing them again to adapt the alignments of their new body position“ (Physiotherapist 4, Female).

### Body Impactors

Changes to the shape and volume of the residual limb are common and are varied with changes to weather and activity levels; even during one physiotherapy session a residual limb can change enough for the socket to become uncomfortable or prevent it from being worn. A once well-fitting socket may become loose, allowing for limb movement in the socket causing residual limb damage. The socket may become so tight, the prosthesis cannot be worn due to pain or skin conditions.

”(Patients) can't get into their socket if they're particularly swollen…I've had a few patients that can't get their limb on for, say, a week at a time” (Physiotherapist 2, Female).

Another issue is that the residual limb gets hot in the prosthesis, due to the fact sockets are typically made of non-breathable materials, with the addition of socks or liners to aid comfort and fit. Temperature build up in the socket is often accompanied by moisture build up due to sweat which can cause further issues for residual limb health (e.g., skin breakdown, infection), but also for prosthesis use.

“As soon as people get sweaty, things change…it causes skin problems and then they can't be on their leg” (Prosthetist 2, Female).

Even when the limb shape and volume have stabilized, the tissue in the residual limb may not be suited to prosthesis use. Adherent scars and skin grafts are not uncommon, particularly for those with traumatic etiology. The internal tissue, that is fused to skeletal structures, can become stiff and immobile, leading to significant pain and skin breakdown, which must be addressed before continued prosthesis use.

“An adherent scar, it sort of sticks when you put a prosthesis on…if the prosthesis is moving over the stump and over the scar itself, and the scar is bound down to the underlying bone; then you can get friction and tissue breakdown” (Physiotherapist 1, Female).

The body's healing process is not easily controlled and is different for each person. Healing can be facilitated by clinicians but is not guaranteed. Open wounds generally prevent the prosthesis being worn, impeding prosthetic rehabilitation and much sought-after progress.

“The soft tissue will mature post-surgery or post-injury, or just after commencing use of a prosthesis; it will mature in its own time…so we have to go at the rate of the body” (Prosthetist 1, Male).

“You can do exercises until you're blue in the face, but you can't actually beat walking” (Physiotherapist 1, Female).

Alongside the physical impactors of the body, the psychology of prosthetic rehabilitation is a crucial factor in success. Individuals are facing a big change in their lives, and many do not want to be impeded by their amputations. It is not uncommon for people to become demotivated when they are not making the progress they hoped.

“There's the psychological aspect of being held back, just when they think they're going to be making progress” (Physiotherapist 1, Female).

### Consequences of Ill-Fit

The socket is a crucial component of the prosthesis and a complex one due to the interface with the body. The direct *consequences of ill-fitting sockets* are focused on the physical health of the residual limb and ability to wear the prosthesis. Due to sockets' rigidity they do not shape to the individual's changes, which can lead to severe pain and discomfort.

“(The socket is) the one factor which is likely to prevent them wearing it entirely, and therefore the comfort is critical to that point” (Prosthetist 1, Male).

Ill-fitting sockets compound issues of residual limb health, facilitating skin breakdown and pressure sore development, which can have lasting impacts on residual limb health, and that of the rest of the body.

“If the prosthesis rubs in any way, shape, or form, then, again, you've got to stop using the prosthesis” (Physiotherapist 1, Female).

Continued prosthesis use with an ill-fitting socket, despite pain or skin conditions, can have adverse effects across the body. Individuals tend to compensate for pain and discomfort by changing their weight distribution or movement. The change can impact the body's biomechanics, potentially leading to musculoskeletal overuse injuries, impacting rehabilitation, function, and long-term health.

### Prosthesis Irritants

Irritations with the prosthesis are not easily solvable due to their make-up and design. The socket is one factor that causes frustration, in particular, its static nature. The rigid nature of the socket means that it does not change with the continual changes to the user's residual limb, which can lead to an ill-fitting socket and associated ramifications.

“(The socket is) only one shape and size at any point. I mean, we can make adjustable sockets now as well, but they're limited. You know, we can't make them change in any way they want to at any point” (Prosthetist 1, Male).

“It's the rigid factor, if you like; that doesn't change as the patient's body changes.” (Physiotherapist 1, Female).

The liners used in some prostheses are also reported to cause issues, particularly around the build-up of heat and sweat in the socket. When liners are paired with socks inside the socket, to increase comfort or minimize movement in the socket, the issues are exacerbated and can lead to residual limb health issues if not dealt with.

“We use a lot of the silicon liners and sealant liners just because you're…enclosed…and they find that even if it's not an hour (exercise) session…they quite often have to stop and take the leg off, dry it down, and then put it back on again” (Physiotherapist 4, Female).

### Limitations of Practice

While fixing known issues may not need significant time, building a rapport between clinician and patient does. The time required for an appointment is often restricted, particularly in the NHS, and so there is not always time to build the clinician-patient relationship, which may mean patients do not feel comfortable talking about personal issues affecting them which may impact their rehabilitation.

“I think it's building your relationship with (patients) as well. But I think that patients in any capacity just seem to want time” (Prosthetist 2, Female).

It was also felt that gaps in support provision contribute to clinicians' time need with patients. Individuals with amputations have undergone a significant life change, requiring physical and emotional support. If the only opportunity for support is during prosthetist or physiotherapist consultations, this adds to the required time.

“Patients don't always get…the mental health input and things like that that they need so sometimes like they will come in and talk to… there's a lot going on for them especially when they're new amputees.” (Prosthetist 2, Female).

When it comes to solving issues, there is no standard practice; e.g., some prefer to remove the prosthesis for wound healing, whereas, others believe it helps to wear it and encourage activity to increase blood-flow and healing for minor wounds. Sometimes, it is unknown what the correct way is to proceed, resulting in a trial and error process.

“It's difficult to know…just by wearing the socket, whether you're going to improve the blood flow to that area, or more typically…worsen the healing rate” (Prosthetist 1, Male).

Similarly, for ill-fitting sockets, increasing the number or thickness of socks is suggested, but specifics cannot be predicted, so mitigation is reactive rather than proactive.

“It's the rigid factor if you like; that doesn't change as the patient's body changes. So, you're constantly playing catch-up” (Physiotherapist 1, Female).

Although patients can be referred back to the physiotherapist by their prosthetist in the UK, this typically happens only when new components have been prescribed. In the NHS, the goal is for individuals to walk and perform daily tasks. However, once initial goals are achieved, there is no standard scheduled check-up. If goals or lifestyles change, individuals will not see a physiotherapist to help them adapt or check good practice.

“(Patients) get up and walking and then that seems to be it really and then it's up to us to then refer them back because if we think they need a bit more but I just don't feel like they get quite enough” (Prosthetist 2, Female).

## Discussion

This study aimed to understand the frustrations and impacts of clinicians through lower-limb prosthetic rehabilitation. Generally in published literature, the views of service users and their carers are sought, and rehabilitation itself is not the specific interest, rather life using a prosthesis is the focus. As a result, this study highlights some of the experiences and impacting factors associated specially with early care of people with amputations.

Analyzing transcripts of the interviewee responses is advantageous to understand the reasoning behind their survey answers; using their own words enhances the impact of their meaning rather than being paraphrased or solely grouped into a statistic.

Five themes were identified in this study: *Service Disparity, Body Impactors, Consequences of Ill-Fit, Prosthesis Irritants*, and *Limitations of Practice*. The individual themes are related closely, with some being the consequences of or exacerbated by others. The identified themes are similar to those identified from the analysis of the prosthesis users' perspectives (7); only the *Limitations of Practice* theme is new and the *Service Disparity* aspect represents a more narrow topic than the *External to Prosthesis* theme identified in the prosthesis user study.

The themes from the current study are also different from the themes identified by Sansam et al. (5), the only other study that evaluated clinician perspectives during lower-limb prosthetic rehabilitation. Given the different focus of the studies, it is expected that there will be variation in the themes identified. Sansam et al. results, however, do highlight issues impacting clinician decision making which may explain some of the issues highlighted in the *Service Disparity* and *Limitations of Practice* themes.

The themes identified in this study align somewhat with previous research that noted socket fit, other qualities of the prosthesis, and prosthetic aftercare to be important (1, 3). One of the most common complaints in the NHS report (4), and analysis of the results from the survey and prosthesis user interviews related to this study (6, 7), was relating to “socket fit,” with specific mention of the lengthy process of socket fitting, with long wait times and multiple trips to hospital required. This importance is reflected in this study's *Consequences of Ill-Fit* theme.

The differences between the results of this analysis compared to previous literature (1, 3–5) are likely due to the focus on rehabilitation in this paper, and that the views are from a clinician perspective and not service users. Prosthesis users give a personal view based on their own experiences, whereas clinicians have a wider perspective which also includes systematic and regulatory knowledge. In particular, the issues related to care provision and early adoption of the prosthesis, which are not present after rehabilitation, are shown in this study. As a result, this study has yielded new insights compared to those previously undertaken. There are, however, several similarities of the themes compared to previous studies evaluating issues experienced by those with limb loss (3) and the assessment of prosthetic care (1), along with the 2018 NHS report on prosthetic services (4) from the perspectives of service users.

*Body Impactors*, such as wound healing time, residual limb volume fluctuation and reactions to wearing a prosthesis (e.g., skin breakdown), can have a significant inhibiting effect on the rehabilitation progress as individuals cannot wear their prosthesis. Whilst other aspects of physiotherapy can continue, such as strength training, gait re-education is often halted if the prosthesis is too painful (e.g., due to adherent scars) or if there is an infection or skin breakdown. The volume change of the residual limb, which can be between 17 and 35% in the 6 months can take around 100 days to stabilize (9, 10). However, the limb also varies throughout the day, depending on temperature and activity levels. The change in volume, and other *Body Impactors*, can lead to an ill-fitting socket and, by extension the *Consequences of Ill-Fit* which have negative impacts on prosthetic rehabilitation.

*Consequences of Ill-Fit* are wide-ranging, but ultimately impact the ability to use the prosthesis. An ill-fitting socket may result in loading of the residual limb in sensitive areas, such as fragile skin tissue or bony prominences. These factors can cause discomfort and pain in the first instance, as well as leading to issues, such as skin breakdown and pressure sores, that have been heavily documented in literature (11–13). Themes of socket fit issues have been identified in previous research also, along with the NHS survey (1, 3, 4).

A socket is fitted to an individual at a static point in time and does not adapt to the changing body, a particular *Prosthesis Irritant*, even adjustable sockets are limited and cannot entirely change shape as required. The Confidence Socket, produced by amparo, a German company, aims to mitigate this issue (14). The socket can be fully remolded to the residual limb in any setting with hot air to allow changes to match that of the residual limb. Their aim is to ensure only one socket is required for the interim stage until a traditional socket is fit. However, whilst this technology means sockets are more easily adaptable to slower residual limb changes, it still does not compensate for any short-term changes.

Prosthesis users may have to wait several months for a new socket to be made for them. *Service Disparity* has highlight that wait times in the public health setting can be long, and that is after the time for a prosthesis user to notice a significant issue rather than routine difficulties. This is a finding supported by the NHS report from 2018 (4).

A key *Limitation of Practice*, is that it is often reactive to issues that occur rather than proactive at mitigating them. It is not uncommon for alternative solutions to ill-fitting sockets to be used, such as the prescription of socks. Prosthetic socks can be used to “cushion” the residual limb to improve user comfort where the prosthetic socket is perceived as too hard, and also compensate for volume reduction. However, this increases the likelihood of temperature and sweat build-up (15). A hot and moist environment is ideal for bacteria and combined with movement of the limb in the socket can lead to skin breakdown and infection (12, 16).

These issues highlight the *Limitations of Practice*, in that care must be reactive to the individual. Bodies will react differently to similar environments; therefore, it is difficult for clinicians to predict specific issues and proactively address them. There is often a trade-off to be made with interventions, with some aspects improving and others increasing in severity.

The clinical setting can exacerbate the difficulties faced by clinicians, and reinforce both *Service Disparity* and *Limitations of Practice*, particularly where the prosthetists are located separately to the physiotherapists. Issues that occur during physiotherapy sessions are more difficult to solve if the prosthetists are not at the same site; prosthesis users may have to wait and travel elsewhere for a solution. The NHS survey of prosthetic services calls for more joint up working between clinicians to try and mitigate these issues (4). A constantly changing clinical team can also hinder the care provided to prosthesis users. With changing team members, more time must be taken to understand histories and interventions already tried.

Issues of *Service Disparity* across the UK were touched upon in previous literature from a clinician perspective (5). It was not identified as a theme in the results, rather an impactor to consider for clinician decision making and a potential explanation for varying views.

There are other aspects that impact prosthetic rehabilitation that are also difficult to control locally. The resources available to teams depend on the setting, for instance the NHS is restricted by national policy for prescriptions [e.g., Microprocessor Controlled Knees (17)] and limited funding compared to private clinics or military. It is noted that time with patients is more limited in the NHS setting, meaning patients are not able to get as much support from their clinicians and have less time to solve issues. However, there is even variation within these groups dependent on locations, the so called “post-code lottery” (18), leading to a *Disparity* in care even before individual circumstances and differences are accounted for.

The majority of issues highlighted by the thematic analysis are well-known to clinicians from their experience or have overlapped with the findings from other studies evaluating prosthetic care from other perspectives (1, 3, 4). However, the current study provides an evidence base of some new issues from clinician perspectives, such as *Service Disparity* and the impacts of staff changes on the continuity of care. Whilst people with amputations are the primary stakeholder of prosthetic rehabilitation, reliant on its outcomes, clinicians have a wide range of experience from which improvements can be made to improve care. The documentation of their perspectives, alongside those of service users, is important to get a full assessment of services.

The current study highlights issues experienced by clinicians during lower-limb prosthetic rehabilitation. The insights from these participants, combined with the documented experiences of prosthesis users, are vital to gain a fuller picture of different aspects of rehabilitation. With the combined understanding of prosthesis user and clinician's views, difficulties can be addressed where possible and communication of expectations can be enhanced.

### Limitations

Participants were recruited for the telephone interviews via a survey that was focused on socket fit. This small, self-select group may have been particularly interested in talking about their issues related to the socket, compared to the overall clinician population. However, the interview results were analyzed in isolation from the survey and the themes based solely on interviewee responses as the survey asked short answer and multiple-choice questions that did not explore the reasoning behind the answers. If more participants from the survey completed an interview, more themes relating to the frustrations and issues during prosthetic rehabilitation may have been identified.

Due to the small sample size, it may be that the results are not representative of the overall clinician population. However, this study provides an initial insight into the experiences of clinicians who work with people with lower-limb amputations through rehabilitation. Most participants worked in an NHS setting, with only two in the private sector. None of the participants were employed by the military, however, it is not uncommon for the military to contract clinicians in these roles. For these reasons, and that participants may have previously worked in a different setting to the one reported and may have varying involvement in decision making processes, it is difficult to fully understand whether this small sample truly represents the full picture of frustrations and issues through lower-limb prosthetic rehabilitation in the UK. There are some professional bodies that encourage the sharing of best practice, which may have influenced some of the views, however, professional body membership of participants was not monitored.

As participant recruitment was limited by the willingness of volunteers, it is unknown whether data saturation was achieved. Therefore, there may be further themes of frustration and issues throughout prosthetic rehabilitation that have not been identified in this study. Different views may also have been captured by defining “frustration” and the period of rehabilitation under consideration in the study, both of which were left to participant interpretation in the current study.

## Conclusion

The thematic analysis gives an insight into the first-hand perspectives of clinicians involved in lower-limb prosthetic rehabilitation. Five related themes have been identified about frustrations that impact rehabilitation: *Service Disparity, Body Impactors, Consequences of Ill-Fit, Prosthesis Irritants*, and *Limitations of Practice*. Many of the issues raised are empirically known amongst clinicians, this study provides formal documentation and a preliminary insight into the clinician perspectives on lower-limb prosthetic rehabilitation. This initial documentation of clinician perspectives provides a foundation for further research and improvements to policy and practice to improve quality of life for people with amputations.

## Data Availability Statement

The datasets presented in this article are not readily available because of ethics restrictions. Requests to access the datasets should be directed to Dr Shruti Turner (s.turner17@imperial.ac.uk).

## Ethics Statement

The studies involving human participants were reviewed and approved by Joint Research and Compliance Office at Imperial College London (ICREC Reference: 18IC4485). The patients/participants provided their written informed consent to participate in this study.

## Author Contributions

ST: study design, data collection, data analysis, and article writing and editing. AB: data analysis, article review, and article editing. AM: supervisor of study, principle investigator, article writing, and article editing. All authors contributed to the article and approved the submitted version.

## Funding

This work was conducted under the auspices of the Royal British Legion Centre for Blast Injury Studies at Imperial College London.

## Conflict of Interest

The authors declare that the research was conducted in the absence of any commercial or financial relationships that could be construed as a potential conflict of interest.

## Publisher's Note

All claims expressed in this article are solely those of the authors and do not necessarily represent those of their affiliated organizations, or those of the publisher, the editors and the reviewers. Any product that may be evaluated in this article, or claim that may be made by its manufacturer, is not guaranteed or endorsed by the publisher.
